# In Vitro Evaluation and Bioinformatics Analysis of Schiff Bases Bearing Pyrazole Scaffold as Bioactive Agents: Antioxidant, Anti-Diabetic, Anti-Alzheimer, and Anti-Arthritic

**DOI:** 10.3390/molecules28207125

**Published:** 2023-10-17

**Authors:** Hamad M. Alkahtani, Abdulrahman A. Almehizia, Mohamed A. Al-Omar, Ahmad J. Obaidullah, Amer A. Zen, Ashraf S. Hassan, Wael M. Aboulthana

**Affiliations:** 1 Department of Pharmaceutical Chemistry, College of Pharmacy, King Saud University, P.O. Box 2457, Riyadh 11451, Saudi Arabia; ahamad@ksu.edu.sa (H.M.A.); mehizia@ksu.edu.sa (A.A.A.); malomar1@ksu.edu.sa (M.A.A.-O.); aobaidullah@ksu.edu.sa (A.J.O.); 2 Chemistry & Forensics Department, Clifton Campus, Nottingham Trent University, Nottingham Ng11 8NS, UK; amer.alhajzen@ntu.ac.uk; 3 Organometallic and Organometalloid Chemistry Department, National Research Centre, Cairo 12622, Egypt; 4 Biochemistry Department, Biotechnology Research Institute, National Research Centre, Cairo 12622, Egypt; wmkamel83@hotmail.com

**Keywords:** Schiff bases, pyrazole scaffold, bioinformatics analysis, anti-arthritic therapeutic, molecular lipophilicity potential, molecular polar surface area

## Abstract

In continuation of our research programs for the discovery, production, and development of the pharmacological activities of molecules for various disease treatments, Schiff bases and pyrazole scaffold have a broad spectrum of activities in biological applications. In this context, this manuscript aims to evaluate and study Schiff base–pyrazole molecules as a new class of antioxidant (total antioxidant capacity, iron-reducing power, scavenging activity against DPPH, and ABTS radicals), anti-diabetic (α-amylase% inhibition), anti-Alzheimer’s (acetylcholinesterase% inhibition), and anti-arthritic (protein denaturation% and proteinase enzyme% inhibitions) therapeutics. Therefore, the Schiff bases bearing pyrazole scaffold (**22a**, **b** and **23a**, **b**) were designed and synthesized for evaluation of their antioxidant, anti-diabetic, anti-Alzheimer’s, and anti-arthritic properties. The results for compound **22b** demonstrated significant antioxidant, anti-diabetic (α-amylase% inhibition), and anti-Alzheimer’s (ACE%) activities, while compound **23a** demonstrated significant anti-arthritic activity. Prediction of in silico bioinformatics analysis (physicochemical properties, bioavailability radar, drug-likeness, and medicinal chemistry) of the target derivatives (**22a**, **b** and **23a**, **b**) was performed. The molecular lipophilicity potential (MLP) of the derivatives **22a**, **b** and **23a**, **b** was measured to determine which parts of the surface are hydrophobic and which are hydrophilic. In addition, the molecular polar surface area (PSA) was measured to determine the polar surface area and the non-polar surface area of the derivatives **22a**, **b** and **23a**, **b**. This study could be useful to help pharmaceutical researchers discover a new series of potent agents that may act as an antioxidant, anti-diabetic, anti-Alzheimer, and anti-arthritic.

## 1. Introduction

**Figure 1:**
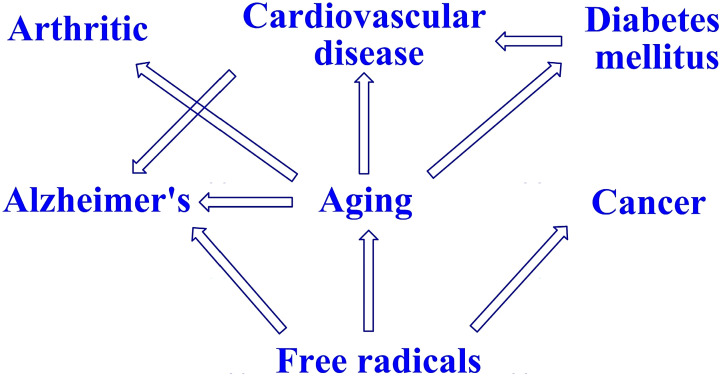
Illustration of the relationship between diverse diseases (diabetes mellitus, Alzheimer, arthritis, and free radicals).

Diabetes is a universal health concern that affects millions of individuals worldwide. Diabetes mellitus is a group of metabolic disorders distinguished by chronic hyperglycemia because of defective production of insulin and/or its activity. Diabetes is classified into type 1 and type 2 diabetes. Diabetes mellitus can cause significant complications such as brain injury, amputations, and heart problems [1]. Alzheimer’s disease (AD) is a form of dementia. The symptoms of Alzheimer’s disease are memory loss and neuronal death. Some of the factors that cause Alzheimer’s disease are cardiovascular disease, old age, and psychosocial factors [2]. Arthritis is an inflammation of the joints of the body. There are various types of arthritis. Osteoarthritis is the most widespread joint disease. The complications of arthritis are pain and disability. The disease of arthritis evolves over decades and ends with the loss of joint function [3]. Free radicals are reactive chemical species and have the ability to attack DNA, proteins, and lipids. Also, free radicals have a relationship with diverse diseases, including cancer, aging, and Alzheimer’s disease. Therefore, antioxidants are very important chemical substances that react with free radicals to prevent the oxidation of biomolecules and protect the body from diseases [4]. Recently, medical research has explored the relationship between diverse diseases, their complications, and their causes [5,6,7]. The complications of free radicals are aging and Alzheimer’s disease. Furthermore, aging may cause diabetes mellitus, Alzheimer’s disease, cardiovascular disease, and arthritis (Figure 1). Therefore, it is important to study the effectiveness of compounds and their biological activities to find a more effective and multi-target drug.

**Figure 2:**
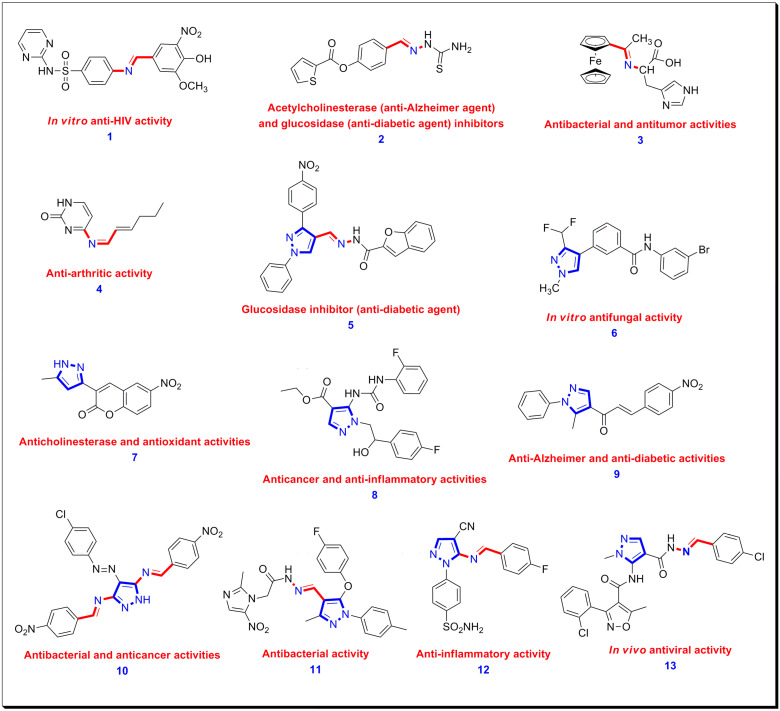
The pharmacological activities of Schiff bases **1**–**4**, the pyrazole scaffold **5**–**9**, and Schiff bases bearing pyrazole scaffold **10**–**13**.

Schiff bases (azomethine compounds) have a spectrum of applications in diverse fields such as the organic, pharmaceutical, and medicinal areas [8]. Schiff bases have a broad range of pharmacological activities, including in vitro anti-HIV activity in, for example, Schiff base **1** (prepared from 5-nitrovaniline and sulphadiazine) [9]. Schiff base **2** is an example of the inhibition activities against acetylcholinesterase (anti-Alzheimer’s agent) and α-glucosidase (anti-diabetic agent) [10]. The ferrocenyl–Schiff base **3** possesses antibacterial and antitumor activities [11]. Schiff base **4,** which is derived from *trans*-2-hexenal with cytosine, is a template for anti-arthritic activity [12] (Figure 2). Furthermore, there are some drugs based on Schiff bases, such as nifuroxazide (an antibiotic for susceptible gastrointestinal infection treatment), thiacetazone (used for tuberculosis infection treatment), and dantrolene (a direct-acting skeletal muscle relaxant) [13].

Pyrazole scaffold has attracted some attention because of its effectiveness and applicability in various fields, primarily the pharmaceutical field [14]. An example of its biological activities is compound **5**, a pyrazole–benzofuran hybrid, that acts as an α-glucosidase inhibitor (anti-diabetic agent) [15]. Pyrazole derivative **6** displays antifungal activity [16]. Coumarin–pyrazole derivative **7** shows good anticholinesterase and antioxidant activities [17]. Ethyl-1*H*-pyrazole-4-carboxylate derivative **8** shows multiple activities as anticancer and anti-inflammatory [18]. Chalcone-engrafted pyrazole **9** shows anti-Alzheimer (acetylcholinesterase% inhibition) and anti-diabetic (α-glucosidase and α-amylase% inhibitions) activities [19] (Figure 2). Also, celecoxib (a non-steroidal anti-inflammatory drug), pyrazofurin (a nucleoside analog related to ribavirin), and ramifenazone (that possesses multiple activities as an analgesic, antipyretic, anti-inflammatory, and antimicrobial) are drugs that have pyrazole scaffold in their structures and are on the market [20].

During the last decade, Schiff bases bearing pyrazole scaffold have attracted growing interest in the pharmaceutical and medicinal fields because of their varied biological effectiveness [21]. Schiff base-bearing pyrazole derivative **10** was shown to be an effective antibacterial for *Salmonella typhimurium* and had anticancer properties against the HeLa cancer cell line [22]. Schiff base-bearing nitroimidazole and pyrazole nuclei **11** demonstrated effective antibacterial activity as an inhibitor against the *E. coli* FabH receptor [23]. Schiff base-bearing pyrazole sulfonamide derivative **12** possesses significant anti-inflammatory activity towards COX-2 enzyme, with IC_50_ equal to 38.73 nM and with a selectivity index equal to 17.47. An in vivo rat paw edema assay of compound **12** was demonstrated to have anti-inflammatory activity, reducing paw edema by up to 42.96% [24]. Schiff base-bearing pyrazole skeleton **13** is an example of in vivo antiviral activity that exhibited protection, inhibition, and therapeutic effects against tobacco mosaic virus (TMV) [25] (Figure 2).

As a result of the foregoing facts about the pharmaceutical and medicinal applications of Schiff bases, pyrazole scaffold, and Schiff bases bearing pyrazole scaffold as well as the importance of multi-target compounds in medicinal chemistry [26,27,28,29,30,31,32,33,34], also, based on the concept of the multi-target drug, and in continuation of our cooperation program [35], thus the goal of the current manuscript is to:

- Synthesize the Schiff bases bearing pyrazole scaffold (**22a**, **b** and **23a**, **b**).
- Evaluation of in vitro antioxidant, anti-diabetic, anti-Alzheimer’s, and anti-arthritic properties.
- Study the in silico bioinformatics analysis (physicochemical properties, the bioavailability radar, drug-likeness, and medicinal chemistry) of Schiff bases bearing pyrazole scaffold (**22a**, **b** and **23a**, **b**).
- Study the molecular lipophilicity potential (MLP) and the molecular polar surface area (PSA) of the derivatives **22a**, **b** and **23a**, **b**.

**Figure 3:**
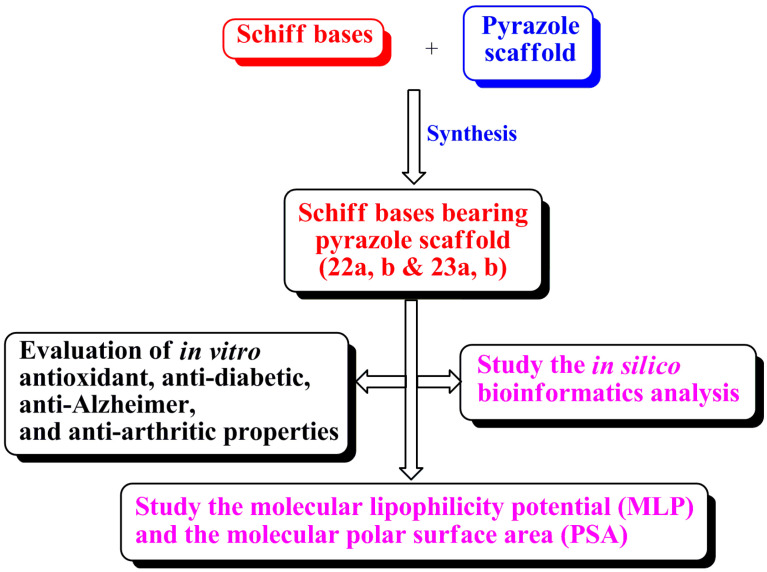
The rationale and the studies of the target compounds, Schiff bases bearing pyrazole scaffold (**22a**, **b** and **23a**, **b**).

Figure 3 illustrates the rationale and the studies.

## 2. Results and Discussion

### 2.1. Chemistry

**Scheme 1:**
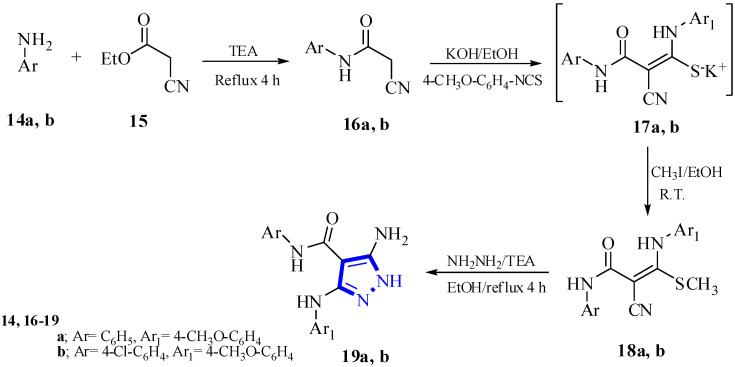
Synthesis of 5-aminopyrazoles **19a, b**.

5-Aminopyrazoles **19a**, **b** [36] as starting materials were produced according to reported procedures and are shown in synthetic Scheme 1.

**Scheme 2:**
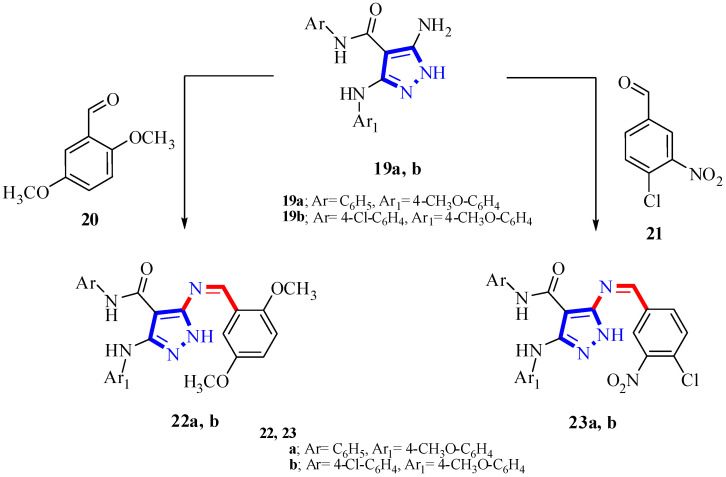
Synthesis of Schiff bases bearing pyrazole scaffold (**22a**, **b** and **23a**, **b**).

The target compounds, Schiff bases bearing pyrazole scaffold (**22a**, **b** and **23a**, **b**), were produced by the one-step reaction strategy, where 5-aminopyrazoles **19a, b** were reacted with 2, 5-dimethoxybenzaldehyde (**20**) or 4-chloro-3-nitrobenzaldehyde (**21**), respectively, in ethanol absolute [37] as represented in synthetic Scheme 2. 

The ^1^H NMR spectra of the target compounds, Schiff bases bearing pyrazole scaffold (**22a**, **b** and **23a**, **b**), were characterized by a single signal around at *δ* = 8.64–8.76 ppm for the –N=CH- proton. Also, they were characterized by three signals around at *δ* = 9.13–9.47, 9.79–10.18, and 11.74–13.01 ppm for the three NH protons which are exchangeable by D_2_O [37] (see Supplementary Material).

### 2.2. In Vitro Biological Activities

Antioxidant, anti-diabetic, anti-Alzheimer’s, and anti-arthritic properties of Schiff bases bearing pyrazole scaffold (**22a**, **b** and **23a**, **b**) were estimated according to the reported methods in the literature [38,39,40].

#### 2.2.1. Antioxidant Activities

The antioxidant activities of the Schiff bases bearing pyrazole scaffold (**22a**, **b** and **23a**, **b**) were evaluated. The results of the total antioxidant capacity (TAC = mg gallic acid/gm), iron-reducing power (IRP = µg/mL), the scavenging activity against DPPH (IC_50_ = µg/mL), and ABTS radicals (%) are listed in Table 1.

Table 1 results show that:

- 5-(2, 5-Dimethoxybenzylideneamino)-3-(4-methoxyphenylamino)-1*H*-pyrazole derivative **22b** exhibited the highest antioxidant activities among all the compounds, which had total antioxidant capacity (TAC) = 42.47 ± 0.09 mg gallic acid/gm, iron-reducing power (IRP) = 24.02 ± 0.05 µg/mL, DPPH radical-scavenging activity (IC_50_ = 13.20 ± 0.03 µg/mL), and ABTS radical-scavenging activity (%) = 35.11 ± 0.08. After compound **22b** in the activity, there was compound **23a**, 5-(4-chloro-3-nitrobenzylideneamino)-3-(4-methoxyphenylamino)-*N*-phenyl-1*H*-pyrazole derivative, which has total antioxidant capacity (TAC) = 36.85 ± 0.08 mg gallic acid/gm, iron-reducing power (IRP) = 20.84 ± 0.05 µg/mL, DPPH radical-scavenging activity (IC_50_ = 15.21 ± 0.03 µg/mL), and ABTS radical-scavenging activity (%) = 30.46 ± 0.07.
- The two compounds **22a** and **23b** have almost the same antioxidant activities (TAC = 34.75 ± 0.08 and 34.56 ± 0.08 mg gallic acid/gm, IRP = 19.65 ± 0.04 and 19.54 ± 0.04 µg/mL, DPPH (IC_50_) = 16.22 ± 0.04 and 16.13 ± 0.04 µg/mL, and ABTS (%) = 28.73 ± 0.06 and 28.56 ± 0.06, respectively).

#### 2.2.2. Anti-Diabetic and Anti-Alzheimer’s Activities

Anti-diabetic and anti-Alzheimer’s activities of all the Schiff bases bearing pyrazole scaffold (**22a**, **b** and **23a**, **b**) were evaluated by figuring out the percentage of the α-amylase and acetylcholinesterase (ACE) inhibitions, respectively. The results of the anti-diabetic and anti-Alzheimer’s activities are detailed in Table 2.

- In the case of α-amylase inhibition (anti-diabetic activity) and using acarbose as the standard reference (% = 69.11 ± 0.15), we observe that compound **22b** showed inhibitor activity of α-amylase (%) = 36.06 ± 0.08, and the next in the activity series is compound **23a** with α-amylase inhibition (%) equal to 31.28 ± 0.07. The two compounds **22a** and **23b** showed almost matching α-amylase inhibition activities equivalent to 29.50 ± 0.06 and 29.34 ± 0.06, respectively.
- In the case of anti-Alzheimer’s activity and using acetylcholinesterase (ACE) inhibition as an indicator for the activity, we observe that compound **22b** showed inhibitor activity of acetylcholinesterase (ACE, %) = 20.71 ± 0.05, and the next is **23a** with percentage inhibition of acetylcholinesterase equal to 17.97 ± 0.04. The inhibitor activities of the two derivatives **22a** and **23b** are almost the same and equal to 16.95 ± 0.04 and 16.85 ± 0.04, respectively.

#### 2.2.3. Anti-Arthritic Activity

The anti-arthritic activities of all the Schiff bases bearing pyrazole scaffold (**22a**, **b** and **23a**, **b**) were evaluated by figuring out the percentage of the protein denaturation inhibition and the proteinase enzyme inhibition using diclofenac sodium as the standard reference. The results of the anti-arthritic activity are listed in Table 2.

- In the case of the protein denaturation inhibition, the more potent compound is **23a** with an inhibitor percentage equal to 22.56 ± 0.05, and then compound **22b** which shows activity equal to 19.57 ± 0.04.
- In the case of the inhibition of proteinase, we also find that compound **23a** (inhibition of proteinase% = 20.71 ± 0.05) is the most active among the Schiff bases bearing pyrazole scaffold, then compound **22b** (inhibition of proteinase% = 17.97 ± 0.04), then the compound **18a** (inhibition of proteinase% = 16.95 ± 0.04), and finally the compound **23b** (inhibition of proteinase% = 16.85 ± 0.04), compared to the standard drug, diclofenac sodium (inhibition of proteinase% = 41.88 ± 0.09). Therefore, the order of activities is diclofenac sodium > **23a** > **22b** > **22a** > **23b**.

**Figure 4:**
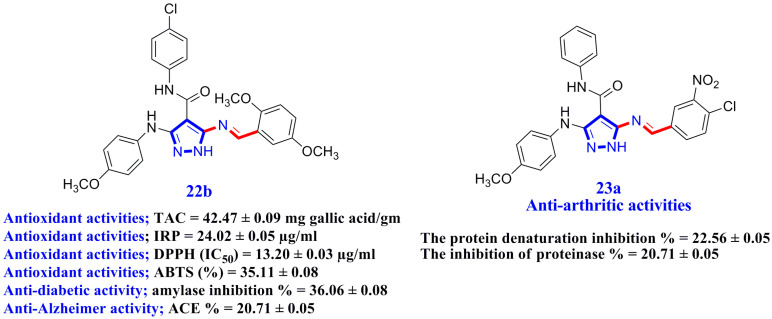
The most potent derivatives **22b** and **23a**.

From the above result of in vitro biological activities studies of Schiff bases bearing pyrazole scaffold **22a**, **b** and **23a**, **b**, we can conclude that the compound **22b** displayed significant antioxidant, anti-diabetic (α-amylase% inhibition), and anti-Alzheimer’s (ACE%) activities, while the compound **23a** displayed significant anti-arthritic activity (Figure 4).

### 2.3. In Silico Bioinformatics Analysis

#### 2.3.1. Physicochemical Properties

Physicochemical properties affect all aspects of drug action because these properties have some biological or chemical effects on the receptors. Also, the physicochemical properties are essential for the successful formulation of the drugs. Specific physicochemical properties shown to be relevant to oral drugs are size, lipophilicity, ionization, hydrogen bonding, polarity, aromaticity, and shape [41]. The physicochemical properties of all the Schiff bases bearing pyrazole scaffold (**22a**, **b** and **23a**, **b**) were yielded using the ADMETlab 2.0 website https://admetmesh.scbdd.com/ (accessed on 11 August 2023)

The physicochemical properties of the Schiff bases bearing pyrazole scaffold (**22a**, **b** and **23a**, **b**) include:

- Molecular weight (MW): sum of the atomic weight values of the atoms in a molecule (optimal: 100~600).
- Volume: van der Waals volume.
- Density: density = MW/volume.
- The number of hydrogen bond acceptors (*n*HA): sum of all O and N (optimal: 0~12).
- The number of hydrogen bond donors (*n*HD): sum of all OHs and NHs (optimal: 0~7).
- The number of rotatable bonds (*n*Rot): (optimal: 0~11).
- Number of rings (*n*Ring): smallest set of smallest rings (optimal: 0~6).
- Number of atoms in the biggest ring (MaxRing): number of atoms involved in the biggest ring (optimal: 0~18).
- Number of heteroatoms (*n*Het): number of non-carbon atoms (hydrogens included, optimal: 1~15).
- The formal charge (fChar) (optimal: −4~4).
- Number of rigid bonds (*n*Rig): number of non-flexible bonds, as opposed to rotatable bonds (optimal: 0~30).
- Flexibility: flexibility = *n*Rot/*n*Rig.
- Stereocenters: number of stereocenters (optimal: ≤2).
- Topological polar surface area (TPSA): sum of tabulated surface contributions of polar fragments (optimal: 0~140).
- logS: the logarithm of aqueous solubility value (optimal: −4~0.5 log mol/L).
- logP: the logarithm of the *n*-octanol/water distribution coefficient (optimal: 0~3 log mol/L).
- logD7.4: the logarithm of the *n*-octanol/water distribution coefficients at pH = 7.4 (optimal: 1~3 log mol/L).

The detailed information on the physicochemical properties of the Schiff bases bearing pyrazole scaffold (**22a**, **b** and **23a**, **b**) can be seen in Table 3.

**Figure 5:**
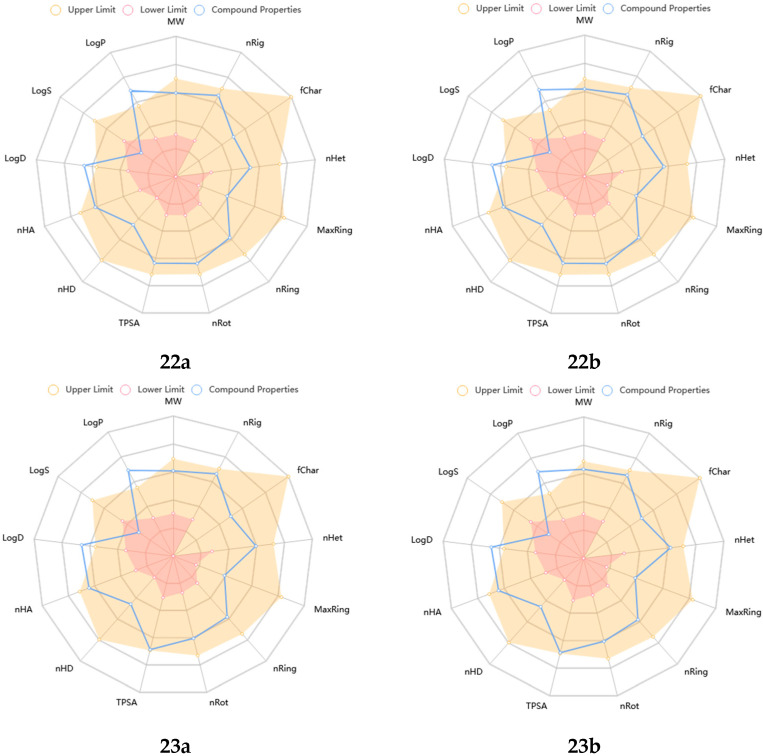
The bioavailability radar of the Schiff bases bearing pyrazole scaffold (**22a**, **b** and **23a**, **b**).

Also, the bioavailability radar of the Schiff bases bearing pyrazole scaffold (**22a**, **b** and **23a**, **b**) for physicochemical properties is shown in Figure 5. The pink area represents the optimal lower limit of physicochemical properties, while the buff area represents the optimal upper limit of physicochemical properties. The blue line represents the physicochemical properties of Schiff bases bearing pyrazole scaffold (**22a**, **b** and **23a**, **b**).

From Figure 5, we can conclude that the blue line of the physicochemical properties of Schiff bases bearing pyrazole scaffold (**22a**, **b** and **23a**, **b**) is in the optimal range between the buff area (the optimal upper limit) and the pink area (the optimal lower limit) except for three properties (logS, logP, and logD7.4) which indicate the solubility property. logS (the logarithm of aqueous solubility value) is less than the optimal lower value. But, logP (the logarithm of the *n*-octanol/water distribution coefficient) and logD7.4 (the logarithm of the *n*-octanol/water distribution coefficients at pH = 7.4) are more than the optimal upper limit. Therefore, we can deduce that these compounds, the Schiff bases bearing pyrazole scaffold (**22a**, **b** and **23a**, **b**), may be orally bioavailable.

#### 2.3.2. Drug-Likeness

Drug-likeness of all the Schiff bases bearing pyrazole scaffold (**22a**, **b** and **23a**, **b**) was predicted using the ADMETlab 2.0 website https://admetmesh.scbdd.com/ (accessed on 11 August 2023).

Detailed information about the drug-likeness of the Schiff bases bearing pyrazole scaffold (**22a**, **b** and **23a**, **b**) can be seen in Table 4.

Drug-likeness was designated based on the physicochemical properties for discovering oral drug candidates [42]. We used three rules, namely the Lipinski rule, the GSK rule, and Pfizer rule, for illustrating drug-likeness.

- Lipinski rule: this rule held four parameters and its requirements are MW ≤ 500, logP ≤ 5, *n*HA ≤ 10, and *n*HD ≤ 5 [43].
- GSK rule: this rule depends on molecular weight (MW) and logP parameters (optimal: MW ≤ 400; logP ≤ 4) [44].
- Pfizer rule: the rule focuses on high logP > 3 and low topological polar surface area (TPSA) < 75 factors [45].

From Table 4, we can conclude that (i) the two derivatives **22a** and **23a** agree with the requirements of the Lipinski rule but the other two derivatives **22b** and **23b** do not match with the Lipinski rule, (ii) all Schiff bases bearing pyrazole scaffold (**22a**, **b** and **23a**, **b**) fulfil the conditions of the Pfizer rule and do not obey the GSK rule.

#### 2.3.3. Medicinal Chemistry

Medicinal chemistry of the Schiff bases bearing pyrazole scaffold (**22a**, **b** and **23a**, **b**) was predicted using the ADMETlab 2.0 website https://admetmesh.scbdd.com/ (accessed on 11 August 2023). 

Medicinal chemistry included the synthetic accessibility score (SA score) and the natural product-likeness score (NP score). Detailed information about the medicinal chemistry of the Schiff bases bearing pyrazole scaffold (**22a**, **b** and **23a**, **b**) can be seen in Table 4.

The synthetic accessibility score (SA score) is designed to estimate the ease of synthesis of drug-like molecules. The SA score ranges from 1 to 10. If the SA score is 1, the compound is easy to synthesize. But, if the SA score is 10 this compound is very difficult to synthesize [46]. According to this rule, the synthetic accessibility score of the Schiff bases bearing pyrazole scaffold (**22a**, **b** and **23a**, **b**) is in the range between 2.8 and 3.0, thus this series is easy to synthesize.

The natural product-likeness score (NP score) is a valuable measure that can help to guide the innovation of new molecules. The NP score ranges from −5 to 5. The higher the score is, the higher the probability that the molecule is like a natural product [47]. According to the rule of the natural product-likeness score, the Schiff bases bearing pyrazole scaffold (**22a**, **b** and **23a**, **b**) are similar and like a natural product as the NP score is between −1.4 and −0.9.

#### 2.3.4. Molecular Lipophilicity Potential (MLP)

The molecular lipophilicity potential (MLP) of all the Schiff bases bearing pyrazole scaffold (**22a**, **b** and **23a**, **b**) was generated using the Molinspiration website https://www.molinspiration.com/cgi-bin/galaxy (accessed on 13 August 2023).

**Figure 6:**
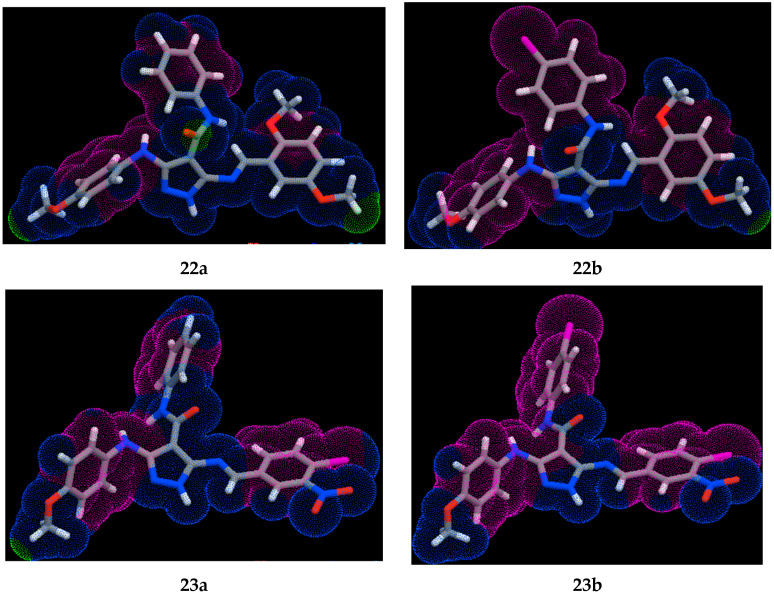
The molecular lipophilicity potential (MLP) of all the Schiff bases bearing pyrazole scaffold (**22a**, **b** and **23a**, **b**).

Molecular lipophilicity potential (MLP) on the molecular surface indicates which parts of the surface are hydrophobic and which are hydrophilic. MLP is calculated from atomic hydrophobicity contributions. The most lipophilic area is marked by a blue color, the intermediate lipophilic area has a pink color, the most hydrophilic area has a yellow color, and the intermediate hydrophilic area has a green color [48]. Figure 6 shows the molecular lipophilicity potential (MLP) of the derivatives **22a**, **b** and **23a, b.**

#### 2.3.5. Molecular Polar Surface Area (PSA)

The molecular polar surface area (PSA) of all the Schiff bases bearing pyrazole scaffold (**22a**, **b** and **23a**, **b**) was generated using the Molinspiration website https://www.molinspiration.com/cgi-bin/galaxy (accessed on 13 August 2023).

**Figure 7:**
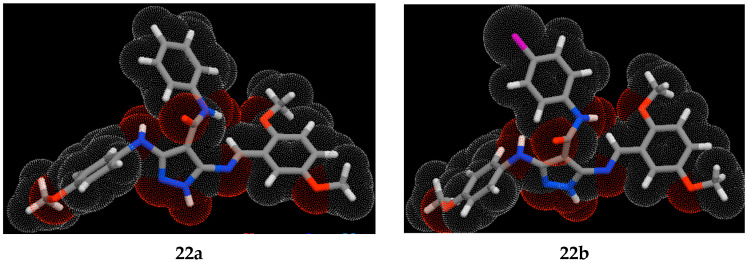

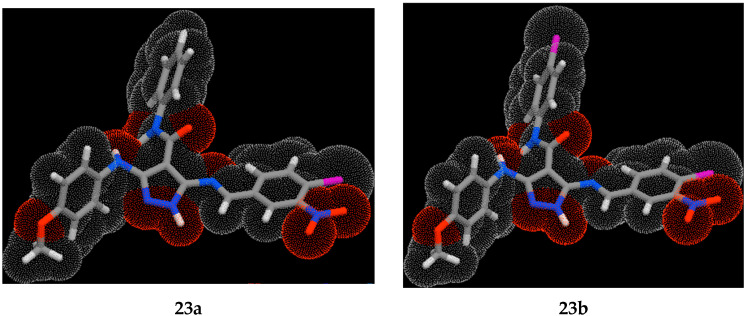
The molecular polar surface area (PSA) of all the Schiff bases bearing pyrazole scaffold (**22a**, **b** and **23a**, **b**).

Molecular polar surface area (PSA) is a very useful parameter for the prediction of drug transport properties. Polar surface area is defined as a sum of surfaces of polar atoms (usually oxygens, nitrogen, and attached hydrogens) in a molecule and it is highlighted by a red color. The non-polar surface area is indicated by a gray-white color [48]. Figure 7 shows the molecular polar surface area (PSA) of the derivatives **22a**, **b** and **23a**, **b**.

## 3. Materials and Methods

### 3.1. Chemistry

5-Amino-1*H*-pyrazoles-4-carboxamides **19a, b** [36] and Schiff bases bearing pyrazole scaffold (**22a**, **b** and **23a**, **b**) [37] were prepared according to the method described in our work.

The spectral data of 5-aminopyrazoles **19a, b** and the target compounds, Schiff bases bearing pyrazole scaffold (**22a**, **b** and **23a**, **b**), are listed in the Supplementary Material.

### 3.2. In Vitro Biological Activities

Antioxidant, anti-diabetic, anti-Alzheimer’s, and anti-arthritic properties of Schiff bases bearing pyrazole scaffold (**22a**, **b** and **23a**, **b**) were estimated according to the reported methods in the literature [38,39,40] (see Supplementary Material).

## 4. Conclusions

In this current manuscript, Schiff bases bearing pyrazole scaffold (**22a**, **b** and **23a**, **b**) were synthesized for evaluation of their in vitro antioxidant, anti-diabetic, anti-Alzheimer’s, and anti-arthritic properties. The result of in vitro biological activities studies of Schiff bases bearing pyrazole scaffold (**22a**, **b** and **23a**, **b**) exhibited that the compound **22b** displayed significant antioxidant (TAC  = 42.47 ± 0.09 mg gallic acid/gm, IRP = 24.02 ± 0.05 µg/mL, DPPH (IC_50_) = 13.20 ± 0.03 µg/mL, and ABTS (%) = 35.11 ± 0.08), anti-diabetic (α-amylase inhibition% = 36.06 ± 0.08), and anti-Alzheimer’s (ACE% = 20.71 ± 0.05) activities, while the compound **23a** displayed significant anti-arthritic activity (the protein denaturation inhibition% = 22.56 ± 0.05 and inhibition of proteinase% = 20.71 ± 0.05). Additionally, the in silico predicted bioinformatics analysis of the target derivatives (**22a**, **b** and **23a**, **b**) revealed that: (i) the compounds (**22a**, **b** and **23a**, **b**) may be orally bioavailable (physicochemical properties analysis). (ii) The two derivatives **22a** and **23a** fulfil the requirements of the Lipinski rule (drug-likeness analysis). (iii) All Schiff bases bearing pyrazole scaffold fulfil the conditions of the Pfizer rule (drug-likeness analysis). (iv) All Schiff bases bearing pyrazole scaffold (**22a**, **b** and **23a**, **b**) are easy to synthesize and like a natural product (medicinal chemistry analysis). Furthermore, molecular lipophilicity potential (MLP) and molecular polar surface area (PSA) studies were performed. These preliminary results of antioxidant, anti-diabetic, anti-Alzheimer’s, and anti-arthritic activities of Schiff bases bearing pyrazole scaffold with in silico bioinformatics analysis could provide excellent models that may guide the discovery of multi-target drugs. In the future, biological activities studies will extend to other structures based on the Schiff bases bearing pyrazole scaffold in the hope of discovering effective and multi-target drugs.

## Figures and Tables

**Table 1:** The antioxidant activities of Schiff bases bearing pyrazole scaffold (**22a**, **b** and **23a**, **b**).

Derivatives	Antioxidant Activity		Scavenging Activity	
	TAC (mg Gallic Acid/gm)	IRP (µg/mL)	DPPH (IC_50_ µg/mL)	ABTS (%)
**22a**	34.75 ± 0.08	19.65 ± 0.04	16.22 ± 0.04	28.73 ± 0.06
**22b**	42.47 ± 0.09 *	24.02 ± 0.05 *	13.20 ± 0.03 *	35.11 ± 0.08 *
**23a**	36.85 ± 0.08	20.84 ± 0.05	15.21 ± 0.03	30.46 ± 0.07
**23b**	34.56 ± 0.08	19.54 ± 0.04	16.13 ± 0.04	28.56 ± 0.06
**STD**	-	-	4.05 ± 0.01	39.09 ± 0.09
			Ascorbic Acid	

* denotes the most effective compound. Values were calculated from three replicates and expressed as mean ± SE.

**Table 2:** The anti-diabetic, anti-Alzheimer’s, and anti-arthritic activities of Schiff bases bearing pyrazole scaffold (**22a**, **b** and **23a**, **b**).

Derivatives	Anti-Diabetic Activity	Anti-Alzheimer’s Activity	Anti-Arthritic Activity	
	α-Amylase Inhibition (%)	Acetylcholinesterase (ACE) Inhibition (%)	Proteinase Denaturation (%)	Inhibition of Proteinase (%)
**22a**	29.50 ± 0.06	16.95 ± 0.04	18.45 ± 0.04	16.95 ± 0.04
**22b**	36.06 ± 0.08 *	20.71 ± 0.05 *	19.57 ± 0.04	17.97 ± 0.04
**23a**	31.28 ± 0.07	17.97 ± 0.04	22.56 ± 0.05 *	20.71 ± 0.05 *
**23b**	29.34 ± 0.06	16.85 ± 0.04	18.35 ± 0.04	16.85 ± 0.04
**STD**	69.11 ± 0.15	-	49.33 ± 0.11	41.88 ± 0.09
	Acarbose		Diclofenac Sodium	

* denotes the most effective compound. Values were calculated from three replicates and expressed as mean ± SE

**Table 3:** The physicochemical properties of Schiff bases bearing pyrazole scaffold (**22a**, **b** and **23a**, **b**).

Derivatives	22a	22b	23a	23b
**Molecular Weight (MW)**	471.190	505.150	490.120	524.080
**Volume**	479.897	495.108	468.876	484.087
**Density**	0.982	1.020	1.045	1.083
***n*** **HA**	9	9	10	10
***n*** **HD**	3	3	3	3
***n*** **Rot**	9	9	8	8
***n*** **Ring**	4	4	4	4
**MaxRing**	6	6	6	6
***n*** **Het**	9	10	11	12
**fChar**	0	0	0	0
***n*** **Rig**	26	26	27	27
**Flexibility**	0.346	0.346	0.296	0.296
**Stereocenters**	0	0	0	0
**TPSA**	113.090	113.090	137.770	137.770
**logS**	−6.608	−7.121	−6.434	−6.807
**logP**	4.421	5.190	4.711	5.455
**logD7.4**	3.783	3.910	3.915	3.807

**Table 4:** The drug-likeness and medicinal chemistry of the Schiff bases bearing pyrazole scaffold (**22a**, **b** and **23a**, **b**).

Derivatives	22a	22b	23a	23b
**Molecular Weight (MW)**	471.190	505.150	490.120	524.080
***n*** **HA**	9	9	10	10
***n*** **HD**	3	3	3	3
**TPSA**	113.090	113.090	137.770	137.770
**logP**	4.421	5.190	4.711	5.455
**Lipinski Rule**	Accepted	Rejected	Accepted	Rejected
**GSK Rule**	Rejected	Rejected	Rejected	Rejected
**Pfizer Rule**	Accepted	Accepted	Accepted	Accepted
**SA Score**	2.886	2.928	2.986	3.027
**NP Score**	−0.903	−1.033	−1.395	−1.448

## Data Availability

Not applicable.

## Supplementary Materials

The following supporting information can be downloaded at: https://www.mdpi.com/article/10.3390/molecules28207125/s1, Spectral data of compounds (**19a**, **b**, **22a**, **b**, and **23a**, **b**) and biological methods.

## References

[1] Priyanka D.N., Harish Prashanth K.V., Tharanathan R.N. (2022). A review on potential anti-diabetic mechanisms of chitosan and its derivatives. Carbohydr. Polym. Technol. Appl..

[2] Lima E., Medeiros J. (2022). Marine Organisms as Alkaloid Biosynthesizers of Potential Anti-Alzheimer Agents. Mar. Drugs.

[3] Laev S.S., Salakhutdinov N.F. (2015). Anti-arthritic agents: Progress and potential. Bioorg. Med. Chem..

[4] Marchi R.C., Campos I.A., Santana V.T., Carlos R.M. (2022). Chemical implications and considerations on techniques used to assess the in vitro antioxidant activity of coordination compounds. Coord. Chem. Rev..

[5] Sadiq I.Z. (2023). Free radicals and oxidative stress: Signaling mechanisms, redox basis for human diseases, and cell cycle regulation. Curr. Mol. Med..

[6] Jena A.B., Samal R.R., Bhol N.K., Duttaroy A.K. (2023). Cellular Red-Ox system in health and disease: The latest update. Biomed. Pharmacother..

[7] Shippy D.C., Ulland T.K. (2023). Lipid metabolism transcriptomics of murine microglia in Alzheimer’s disease and neuroinflammation. Sci. Rep..

[8] Egu S.A., Ali I., Khan K.M., Chigurupati S., Qureshi U., Salar U., Taha M., Felemban S.G., Venugopal V., Ul-Haq Z. (2022). Syntheses, in vitro, and in silico studies of rhodanine-based schiff bases as potential α-amylase inhibitors and radicals (DPPH and ABTS) scavengers. Mol. Divers..

[9] Elangovan N., Thomas R., Sowrirajan S., Manoj K.P., Irfan A. (2022). Synthesis, Spectral Characterization, Electronic Structure and Biological Activity Screening of the Schiff Base 4-((4-Hydroxy-3-Methoxy-5-Nitrobenzylidene) Amino)-*N*-(Pyrimidin-2-yl) Benzene Sulfonamide from 5-Nitrovaniline and Sulphadiazene. Polycycl. Aromat. Compd..

[10] Tokalı F.S., Taslimi P., Usanmaz H., Karaman M., Şendil K. (2021). Synthesis, characterization, biological activity and molecular docking studies of novel schiff bases derived from thiosemicarbazide: Biochemical and computational approach. J. Mol. Struct..

[11] Sayed F.N., Mohamed G.G. (2022). Newly synthesized lanthanides complexes of ferrocene-based Schiff base with high biological activities and improved molecular docking data. J. Organomet. Chem..

[12] Surendar P., Pooventhiran T., Rajam S., Bhattacharyya U., Bakht M.A., Thomas R. (2021). Quasi liquid Schiff bases from trans-2-hexenal and cytosine and l-leucine with potential antieczematic and antiarthritic activities: Synthesis, structure and quantum mechanical studies. J. Mol. Liq..

[13] Said M.A., Khan D.J., Al-Blewi F.F., Al-Kaff N.S., Ali A.A., Rezki N., Aouad M.R., Hagar M. (2021). New 1, 2, 3-Triazole scaffold schiff bases as potential anti-COVID-19: Design, synthesis, DFT-molecular docking, and cytotoxicity aspects. Vaccines.

[14] Islam M.S., Al-Majid A.M., Sholkamy E.N., Yousuf S., Ayaz M., Nawaz A., Wadood A., Rehman A.U., Verma V.P., Motiur Rahman A.F.M. (2022). Synthesis of Spiro-oxindole Analogs Engrafted Pyrazole Scaffold as Potential Alzheimer’s Disease Therapeutics: Anti-oxidant, Enzyme Inhibitory and Molecular Docking Approaches. ChemistrySelect.

[15] Azimi F., Azizian H., Najafi M., Khodarahmi G., Saghaei L., Hassanzadeh M., Ghasemi J.B., Faramarzi M.A., Larijani B., Hassanzadeh F. (2021). Design, synthesis, biological evaluation, and molecular modeling studies of pyrazole-benzofuran hybrids as new α-glucosidase inhibitor. Sci. Rep..

[16] Yu B., Zhao B., Hao Z., Chen L., Cao L., Guo X., Zhang N., Yang D., Tang L., Fan Z. (2021). Design, synthesis and biological evaluation of pyrazole-aromatic containing carboxamides as potent SDH inhibitors. Eur. J. Med. Chem..

[17] Benazzouz-Touami A., Chouh A., Halit S., Terrachet-Bouaziz S., Makhloufi-Chebli M., Ighil-Ahriz K., Silva A.M. (2022). New Coumarin-Pyrazole hybrids: Synthesis, Docking studies and Biological evaluation as potential cholinesterase inhibitors. J. Mol. Struct..

[18] Signorello M.G., Rapetti F., Meta E., Sidibè A., Bruno O., Brullo C. (2021). New series of pyrazoles and imidazo-pyrazoles targeting different cancer and inflammation pathways. Molecules.

[19] Islam M.S., Al-Majid A.M., Sholkamy E.N., Yousuf S., Ayaz M., Nawaz A., Wadood A., Rehman A.U., Verma V.P., Bari A. (2022). Synthesis, molecular docking and enzyme inhibitory approaches of some new chalcones engrafted pyrazole as potential antialzheimer, antidiabetic and antioxidant agents. J. Mol. Struct..

[20] Eweas A.F., El-Nezhawy A.O.H., Abdel-Rahman R.F., Baiuomy A.R. (2015). Design, Synthesis. Vivo Anti-inflammatory, Analgesic Activities and Molecular Docking of Some Novel Pyrazolone Derivatives. Med. Chem..

[21] Al-Ghorbani M., Alharbi O., Al-Odayni A.B., Abduh N.A. (2023). Quinoline-and Isoindoline-Integrated Polycyclic Compounds as Antioxidant, and Antidiabetic Agents Targeting the Dual Inhibition of α-Glycosidase and α-Amylase Enzymes. Pharmaceuticals.

[22] Şener N., Özkinali S., Altunoglu Y.C., Yerlikaya S., Gökçe H., Zurnaci M., Gür M., Baloglu M.C., Şener İ. (2021). Antiproliferative properties and structural analysis of newly synthesized Schiff bases bearing pyrazole derivatives and molecular docking studies. J. Mol. Struct..

[23] Sangani C.B., Makwana J.A., Duan Y.T., Tarpada U.P., Patel Y.S., Patel K.B., Dave V.N., Zhu H.L. (2015). Design, synthesis, and antibacterial evaluation of new Schiff’s base derivatives bearing nitroimidazole and pyrazole nuclei as potent *E. coli* FabH inhibitors. Res. Chem. Intermed..

[24] Sharma S., Kumar D., Singh G., Monga V., Kumar B. (2020). Recent advancements in the development of heterocyclic anti-inflammatory agents. Eur. J. Med. Chem..

[25] Sharma P.C., Sharma D., Sharma A., Saini N., Goyal R., Ola M., Chawla R., Thakur V.K. (2020). Hydrazone comprising compounds as promising anti-infective agents: Chemistry and structure-property relationship. Mater. Today Chem..

[26] Noce B., Di Bello E., Fioravanti R., Mai A. (2023). LSD1 inhibitors for cancer treatment: Focus on multi-target agents and compounds in clinical trials. Front. Pharmacol..

[27] Alamri M.A. (2023). Bioinformatics and network pharmacology-based study to elucidate the multi-target pharmacological mechanism of the indigenous plants of Medina valley in treating HCV-related hepatocellular carcinoma. Saudi Pharm. J..

[28] Al-Wahaibi L.H., Mohammed A.F., Abdelrahman M.H., Trembleau L., Youssif B.G. (2023). Design, Synthesis, and Biological Evaluation of Indole-2-carboxamides as Potential Multi-Target Antiproliferative Agents. Pharmaceuticals.

[29] Kumari M., Waseem M., Subbarao N. (2023). Discovery of multi-target mur enzymes inhibitors with anti-mycobacterial activity through a Scaffold approach. J. Biomol. Struct. Dyn..

[30] Ragab M.A., Eldehna W.M., Nocentini A., Bonardi A., Okda H.E., Elgendy B., Ibrahim T.S., Abd-Alhaseeb M.M., Gratteri P., Supuran C.T. (2023). 4-(5-Amino-pyrazol-1-yl) benzenesulfonamide derivatives as novel multi-target anti-inflammatory agents endowed with inhibitory activity against COX-2, 5-LOX and carbonic anhydrase: Design, synthesis, and biological assessments. Eur. J. Med. Chem..

[31] Nandi S., Chauhan B., Tarannum H., Khede M.K. (2023). Multi-target polypharmacology of 4-aminoquinoline compounds against malaria, tuberculosis and cancer. Curr. Top. Med. Chem..

[32] Halder A.K., Mitra S., Cordeiro M.N.D. (2023). Designing multi-target drugs for the treatment of major depressive disorder. Expert Opin. Drug Discov..

[33] Sadek K.U., Mekheimer R.A., Abd-Elmonem M., Abo-Elsoud F.A., Hayallah A.M., Mostafa S.M., Abdellattif M.H., Abourehab M.A., Farghaly T.A., Elkamhawy A. (2023). Recent Developments in the Synthesis of Hybrid Heterocycles, A Promising Approach to Develop Multi-target Antibacterial Agents. J. Mol. Struct..

[34] Liu Q., Zhang B., Wang Y., Wang X., Gou S. (2022). Discovery of phthalazino [1,2-*b*]quinazolinone derivatives as multi-target HDAC inhibitors for the treatment of hepatocellular carcinoma via activating the p53 signal pathway. Eur. J. Med. Chem..

[35] Hassan A.S. (2022). Antimicrobial evaluation, in silico ADMET prediction, molecular docking, and molecular electrostatic potential of pyrazole-isatin and pyrazole-indole hybrid molecules. J. Iran. Chem. Soc..

[36] Khatab T.K., Hassan A.S., Hafez T.S. (2019). V_2_O_5_/SiO_2_ as an efficient catalyst in the synthesis of 5-amino-pyrazole derivatives under solvent free condition. Bull. Chem. Soc. Ethiop..

[37] Hassan A.S., Morsy N.M., Awad H.M., Ragab A. (2022). Synthesis, molecular docking, and in silico ADME prediction of some fused pyrazolo [1,5-*a*]pyrimidine and pyrazole derivatives as potential antimicrobial agents. J. Iran. Chem. Soc..

[38] Aboulthana W.M., Omar N.I., El-Feky A.M., Hasan E.A., Ibrahim N.E.S., Youssef A.M. (2022). In vitro study on effect of zinc oxide nanoparticles on the biological activities of croton *Tiglium* L. Seeds extracts. Asian Pac. J. Cancer Prev..

[39] Azeem M.N.A., Ahmed O.M., Shaban M., Elsayed K.N. (2022). In vitro antioxidant, anticancer, anti-inflammatory, anti-diabetic and anti-Alzheimer potentials of innovative macroalgae bio-capped silver nanoparticles. Environ. Sci. Pollut. Res..

[40] Al-Radadi N.S., Faisal S., Alotaibi A., Ullah R., Hussain T., Rizwan M., Zaman N., Iqbal M., Iqbal A., Ali Z. (2022). Zingiber officinale driven bioproduction of ZnO nanoparticles and their anti-inflammatory, anti-diabetic, anti-Alzheimer, anti-oxidant, and anti-microbial applications. Inorg. Chem. Commun..

[41] Xiong G., Wu Z., Yi J., Fu L., Yang Z., Hsieh C., Yin M., Zeng X., Wu C., Lu A. (2021). ADMETlab 2.0: An integrated online platform for accurate and comprehensive predictions of ADMET properties. Nucleic Acids Res..

[42] Daina A., Michielin O., Zoete V. (2017). SwissADME: A free web tool to evaluate pharmacokinetics, drug-likeness and medicinal chemistry friendliness of small molecules. Sci. Rep..

[43] Lipinski C.A., Lombardo F., Dominy B.W., Feeney P.J. (2001). Experimental and computational approaches to estimate solubility and permeability in drug discovery and development settings. Adv. Drug Deliv. Rev..

[44] Gleeson M.P. (2008). Generation of a set of simple, interpretable ADMET rules of thumb. J. Med. Chem..

[45] Hughes J.D., Blagg J., Price D.A., Bailey S., DeCrescenzo G.A., Devraj R.V., Ellsworth E., Fobian Y.M., Gibbs M.E., Gilles R.W. (2008). Physiochemical drug properties associated with in vivo toxicological outcomes. Bioorg. Med. Chem. Lett..

[46] Ertl P., Schuffenhauer A. (2009). Estimation of synthetic accessibility score of drug-like molecules based on molecular complexity and fragment contributions. J. Cheminform..

[47] Ertl P., Roggo S., Schuffenhauer A. (2008). Natural product-likeness score and its application for prioritization of compound libraries. J. Chem. Inf. Model.

[48] Alam M.S., Lee D.U., Bari M.L. (2014). Antibacterial and cytotoxic activities of Schiff base analogues of 4-aminoantipyrine. J. Korean Soc. Appl. Biol. Chem..

