# Supplementary material for: In Vitro Evaluation and Bioinformatics Analysis of Schiff Bases Bearing Pyrazole Scaffold as Bioactive Agents: Antioxidant, Anti-Diabetic, Anti-Alzheimer, and Anti-Arthritic
Source: Molecules. 2023 Oct 17;28(20):7125. doi: 10.3390/molecules28207125 (PMC10609138; doi:10.3390/molecules28207125)
Supplement: Supplementary file 1 [file molecules-28-07125-s001.zip › molecules-2625361-supplementary.pdf]

SUPPLEMENTARY MATERIAL

For

In Vitro Evaluation and Bioinformatics Analysis of Schiff Bases Bearing Pyrazole Scaffold as Bioactive Agents: Antioxidant, Anti-Diabetic, Anti-Alzheimer, and Anti-Arthritic

Hamad M. Alkahtani[1], Abdulrahman A. Almehizia[1]., Mohamed A. Al-Omar[1], Ahmad J. Obaidullah[1], Amer A. Zen[2], Ashraf S. Hassan[3],* and Wael M. Aboulthana[4]

[1] Department of Pharmaceutical Chemistry, College of Pharmacy, King Saud University, P.O. Box 2457, Riyadh 11451, Saudi Arabia; ahamad@ksu.edu.sa (H.M.A.); mehizia@ksu.edu.sa (A.A.A.); malomar1@ksu.edu.sa (M.A.A.-O.); aobaidullah@ksu.edu.sa (A.J.O.)

[2] Chemistry & Forensics Department, Clifton Campus, Nottingham Trent University, Nottingham Ng11 8NS, UK; amer.alhajzen@ntu.ac.uk (A.A.Z.)

[3] Organometallic and Organometalloid Chemistry Department, National Research Centre, Dokki 12622, Cairo, Egypt

[4] Biochemistry Department, Biotechnology Research Institute, National Research Centre, Dokki 12622, Cairo, Egypt; wmkamel83@hotmail.com (W.M.A.)

* Correspondence: ashraf_salmoon@yahoo.com; Tel.: +20-100-664-5444 (A.S.H.)

Chemicals and Apparatus

All melting points were measured on a Gallenkamp melting point apparatus and are uncorrected. The IR spectra were recorded (KBr disk) on a PerkinElmer 1650 FTIR instrument. ^{1}H NMR (500 MHz) and ^{13}C NMR (125 MHz) spectra were recorded on a JEOL spectrometer using DMSO-d_6 as solvent and tetramethylsilane (TMS) as an internal standard. Chemical shifts (δ-values) are reported in ppm and coupling constants (J) in Hz.

Reagents and solvents used in the synthesis were purchased from Sigma-Aldrich. 2, 5-Dimethoxybenzaldehyde (**20**) and 4-chloro-3-nitrobenzaldehyde (**21**) were of Merck AR grade, Germany.

Synthesis of 5-amino-*N*-aryl-3-(4-methoxyphenylamino)-1*H*-pyrazole-4-carboxamide (19a, b)

A mixture of *N*-substituted cyanoacetamide derivatives **16a, b** (0.01 mol, which was prepared by the reaction of aromatic amines **14a, b** with ethyl cyanoacetate (**15**)) and 4-methoxyphenylisothiocyanate (0.01 mol) was heated for 5-10 min in ethanol (25 ml) containing potassium hydroxide (0.01 mol). After cooling, methyl iodide (0.01 mol) was added. The reaction mixture was stirred at room temperature for 1 h and then poured onto ice water. The precipitated product was filtered and recrystallized from ethanol to form **18a, b**.

A mixture of compounds **18a, b** (0.01 mol), hydrazine hydrate (0.01 mol), and few drops of triethylamine in ethanol (30 ml) was refluxed for 4 h and then the solvent evaporated under reduced pressure. The resulting solid product was collected by filtration and recrystallized from ethanol.

5-Amino-3-(4-methoxyphenylamino)-*N*-phenyl-1*H*-pyrazole-4-carboxamide (19a)

White crystals; M.p.: 175-177 °C; ^{1}H NMR (DMSO-d_6, δ/ppm): 3.63 (s, 3H, OCH_3), 5.98 (s, 2H, NH_2, D_2O exchangeable), 6.76 (d, 2H, aromatic, J_{HH}=7.7 Hz), 6.98 (t, 1H, aromatic, J_{HH}=7.7 Hz), 7.18 (t, 2H, aromatic, J_{HH}=8.4 Hz), 7.25 (d, 2H, aromatic, J_{HH}=7.7 Hz), 7.45 (d, 2H, aromatic, J_{HH}=7.7 Hz), 8.30 (s, 1H, NH, D_2O exchangeable), 8.74 (s, 1H, NH, D_2O exchangeable), 11.22 (s, 1H, NH, D_2O exchangeable); ^{13}C NMR (DMSO-d_6, δ/ppm) 55.6 (-OCH_3), 88.3 (C_4, pyrazole), 114.7, 117.5, 120.3, 123.3, 129.1, 137.6, 139.4 (11C, aromatic), 149.1 (C_5, pyrazole), 150.9 (C_3, pyrazole), 153.1 (C, aromatic), 163.6 (C=O).

5-amino-N-(4-chlorophenyl)-3-(4-methoxyphenylamino)-1H-pyrazole-4-carboxamide (19b)

White crystals; M.p.: 190-192 °C; ^{1}H NMR (DMSO-d_6, δ/ppm): 3.64 (s, 3H, OCH_3), 6.00 (s, 2H, NH_2, D_2O exchangeable), 6.77-7.51 (m, 8H, aromatic), 8.30 (s, 1H, NH, D_2O exchangeable), 8.82 (s, 1H, NH, D_2O exchangeable), 11.20 (s, 1H, NH, D_2O exchangeable); ^{13}C NMR (DMSO-d_6, δ/ppm) 55.7 (-OCH_3), 87.9 (C_4, pyrazole), 114.7, 117.7, 121.9, 126.8, 128.9, 137.4, 138.4 (11C, aromatic), 149.1 (C_5, pyrazole), 151.1 (C_3, pyrazole), 153.2 (C, aromatic), 163.6 (C=O).

Synthesis of Schiff bases 22a, b and 23a, b

A mixture of 5-amino-*N*-aryl-1*H*-pyrazole-4-carboxamide derivatives **19a**, **b** (0.01 mol) and various aldehydes (0.01 mol) {namely: 2,5-dimethoxybenzaldehyde (**20**) or 4-chloro-3-nitrobenzaldehyde (**21**)} with a catalytic amount of glacial acetic acid (1 mL) in absolute ethanol (25 mL) was refluxed for 2 hours and then left to cool. The solid product was filtered off, dried and finally recrystallized from ethanol to afford the corresponding Schiff bases **22a, b and 23a, b**.

5-(2,5-Dimethoxybenzylideneamino)-3-(4-methoxyphenylamino)-*N*-phenyl-1*H*-pyrazole-4-carboxamide (22a)

Orange crystals; M.p.: 234-236 °C; Yield (69%); IR (KBr) ν_{max}/cm^{-1} 3389, 3320 (3NH), 1654 (C=O), 1594 (C=N), 1552 (C=C); ^{1}H NMR (DMSO-d_6, δ/ppm): 3.72, 3.83, 3.89 (3s, 9H, 3OCH_3), 6.91 (d, 2H, J = 8.0 Hz, *p*-methoxyphenyl ring), 7.08-7.35 (m, 7H, Ar-H), 7.50 (s, 1H, 2,5-dimethoxyphenyl ring), 7.65 (d, 2H, J = 8.3 Hz, *p*-methoxyphenyl ring), 8.69 (s, 1H, -CH=N-), 9.47, 10.18, 13.01 (3s, 3H, 3NH exchangeable by D_2O); Anal. Calcd. (%) for $C_{26}H_{25}N_5O_4$ (471.51): C, 66.23; H, 5.34; N, 14.85. Found: C, 66.62; H, 5.17; N, 14.68 %.

N-(4-Chlorophenyl)-5-(2,5-dimethoxybenzylideneamino)-3-(4-methoxyphenylamino)-1H-pyrazole-4-carboxamide (22b)

Orange crystals; M.p.: 250-252 °C; Yield (74%); IR (KBr) ν_{max}/cm^{-1} 3432, 3251 (3NH), 1653 (C=O), 1597 (C=N), 1544 (C=C); ^1H NMR (DMSO-d_6, δ/ppm): 3.73, 3.82, 3.88 (3s, 9H, $3OCH_3$), 6.89, 6.91 (dd, 2H, J = 2.8 & 9.5 Hz, *p*-methoxyphenyl ring), 7.18 (d, 1H, J = 2.8 Hz, 2,5-dimethoxyphenyl ring), 7.21 (d, 1H, J = 2.7 Hz, 2,5-dimethoxyphenyl ring), 7.38 (d, 2H, J = 9.2 Hz, *p*-chlorophenyl ring), 7.42 (d, 2H, J = 9.0 Hz, *p*-chlorophenyl ring), 7.61 (s, 1H, 2,5-dimethoxyphenyl ring), 7.66, 7.68 (dd, 2H, J = 9.2, 2.7 Hz, *p*-methoxyphenyl ring), 8.64 (s, 1H, -CH=N-), 9.34, 10.13, 11.74 (3s, 3H, 3NH exchangeable by D_2O); ^{13}C NMR (DMSO-d_6, δ/ppm): 55.26, 55.59, 56.47 (3C, $3OCH_3$), 92.55, 109.44, 114.21, 114.42, 119.00, 120.58, 121.94, 123.30, 126.66, 128.83, 134.18, 137.62, 153.41, 153.81, 154.87, 156.70, 156.90, 160.72, 162.88 (23C); Anal. Calcd. (%) for $C_{26}H_{24}ClN_5O_4$ (505.95): C, 61.72; H, 4.78; N, 13.84; Found: C, 61.90; H, 4.51; N, 13.98 %.

5-(4-Chloro-3-nitrobenzylideneamino)-3-(4-methoxyphenylamino)-N-phenyl-1H-pyrazole-4-carboxamide (23a)

Buff crystals; M.p.: 252-254 °C; Yield (72%); IR (KBr) ν_{max}/cm^{-1} 3431, 3235 (3NH), 1653 (C=O), 1615 (C=N), 1513 (C=C); ^1H NMR (DMSO-d_6, δ/ppm): 3.70 (s, 3H, OCH_3), 6.89 (d, 2H, J = 8.3 Hz, *p*-methoxyphenyl ring), 7.06 (t, 1H, J = 7.3 Hz, phenyl ring), 7.31-7.34 (m, 4H, phenyl ring), 7.67 (d, 2H, J = 7.9 Hz, *p*-methoxyphenyl ring), 8.00 (d, 1H, J = 8.3, 4-chloro-3-nitrophenyl ring), 8.30 (d, 1H, J = 8.3, 4-chloro-3-nitrophenyl ring), 8.67 (s, 1H, 4-chloro-3-nitrophenyl ring), 8.76 (s, 1H, -CH=N-), 9.13, 9.79, 12.80 (3s, 3H, 3NH exchangeable by D_2O); ^{13}C NMR (DMSO-d_6, δ/ppm): 55.23 (1C, OCH_3), 96.78, 114.61, 118.92, 119.73, 121.51, 123.06, 125.26, 126.27, 128.87, 132.72, 133.81, 134.09, 138.39, 147.84, 152.02, 153.37, 157.62, 160.86, 162.42 (23C); Anal. Calcd. (%) for $C_{24}H_{19}ClN_6O_4$ (490.90): C, 58.72; H, 3.90; N, 17.12; Found: C, 58.99; H, 3.79; N, 17.28 %.

5-(4-Chloro-3-nitrobenzylideneamino)-N-(4-chlorophenyl)-3-(4-methoxyphenylamino)-1H-pyrazole-4-carboxamide (23b)

Yellow crystals; M.p.: 280-282 °C; yield (71%); IR (KBr) ν_{max}/cm^{-1} 3440, 3280, 3230 (3NH), 1653 (C=O), 1615 (C=N), 1561 (C=C); ^1H NMR (DMSO-d_6, δ/ppm): 3.72 (s, 3H, OCH_3), 6.89 (d, 2H, J = 8.7 Hz, *p*-methoxyphenyl ring), 7.35 (d, 4H, J = 8.8 Hz, *p*-chlorophenyl ring), 7.70 (d, 2H, J = 8.8 Hz, *p*-methoxyphenyl ring), 7.96 (d, 1H, J = 8.4 Hz, 4-chloro-3-nitrophenyl ring), 8.27 (d, 1H, J = 8.3 Hz, 4-chloro-3-nitrophenyl ring), 8.26 (s, 1H, 4-chloro-3-nitrophenyl ring), 8.72 (s, 1H, -CH=N-), 9.14, 9.87, 12.94 (3s, 3H, 3NH exchangeable by D_2O); Anal. Calcd. (%) for $C_{24}H_{18}Cl_2N_6O_4$ (525.34): C, 54.87; H, 3.45; N, 16.00; Found: C, 55.05; H, 3.28; N, 16.19 %.

In vitro biological activities

Antioxidant activity

Total antioxidant capacity

Total antioxidant capacity (TAC) was determined by evaluating the green phosphate/Mo^{5+} complex at wavelength 695 nm using the method described by Prieto *et al*. It was expressed as mg gallic acid equivalent per gram dry weight. Briefly, 0.1 ml of the extract was combined with 1 ml of reagent solution (0.3 N sulfuric acid, 28 mM sodium phosphate and 4 mM ammonium molybdate). The tubes were capped and incubated in a boiling water bath for 90 min. Then, the samples were cooled to room temperature and the absorbance was measured at wave length 695 nm against blank containing methanol (80%) instead of extract. Antioxidant capacity was expressed as mg gallic acid equivalent per gram dry weight.

Iron-reducing power

Total iron-reducing power was assessed using the method suggested by Oyaizu as μg/mL. A high absorbance of the reaction mixture at 700 nm indicates a higher reducing power. In brief, 1ml of the

extract (1mg/ml) was mixed with 1ml of 200 mM of sodium phosphate buffer (pH 6.6) and 1ml of 1% potassium ferricyanide. Consequently, the mixture was incubated at 50 °C for 20 min. followed by adding 1ml of trichloroacetic acid (10% w/v). The mixture was centrifuged at 2000 rpm for 10 min. The upper layer solution (2.5 ml) was mixed with 2.5 ml of double deionised water and 1ml of freshly prepared ferric chloride solution (0.1%). The absorbance was measured at 700 nm against blank prepared without adding extract. Various concentrations of ascorbic acid were used as standard. A high absorbance of the reaction mixture at 700 nm indicates a higher reducing power.

DPPH radical-scavenging activity

The scavenging activity against 1, 1-diphenyl-2-picryl-hydrazyl (DPPH) radical was assayed by calculating the median inhibitory concentration (IC_{50}) according to the method suggested. The substance (antioxidant) that can donate a hydrogen atom to solution containing DPPH- can reduce the stable free radical and change the color of solution from violet to pale yellow. Non reacted DPPH-radical form was quantified by measuring intensity of a light-purple colored DPPH methanol solution in the visible range at 518 nm against blank solution by spectroscopic method.

Then, the inhibition % was plotted against concentration, and from the graph IC_{50} was calculated.

Percent inhibition of the DPPH free radical was calculated by the following equation:

The DPPH radical scavenging activity %:

$$\text{(Control OD} - \text{sample OD/control OD)} \times 100$$

Serial concentrations spanning from 0.78 till 100 μg/mL were prepared from different samples. Two milliliter of DPPH solution (100 μM) in ethanol was mixed with 2 mL of sample (100μg/mL). The effective test concentrations of DPPH and the sample were 50μM and 50μg/mL, respectively. The reaction mixture of different dilutions, for different concentrations, was vortexed thoroughly then incubated in the dark at room temperature for 30 min. Thereafter, the absorbance was measured spectrophotometrically at 518 nm against blank (ethanol). For control, 2 mL of ethanol was added instead of sample and run simultaneously with the test. Ascorbic acid was used as positive control.

ABTS radical scavenging assay

The 2, 2'-azinobis-(3-ethylbenzothiazoline-6-sulfonic acid) (ABTS) assay was carried out to determine the percent of the ABTS radical inhibition (%) according to the method modified by Arnao *et al* using ascorbic acid as standard. The stock solutions were consisting of ABTS solution (7 mM) and potassium persulfate solution (2.4 mM). Then, the working solution was prepared by mixing the two stock solutions in equal quantities and allowing them to react at room temperature in dark place for 14 h. The solution was then diluted by mixing 1 ml ABTS solution with 60 ml methanol to obtain an absorbance of 0.706 ± 0.01 units at 734 nm using a spectrophotometer. Fresh ABTS solution was prepared for each assay. The extracts (1 ml) were allowed to react with 1 ml of the ABTS solution and the absorbance was taken at 734 nm after 7 min using a spectrophotometer. The ABTS scavenging capacity of the extract was compared with that of ascorbic acid

Anti-diabetic activity (α-amylase inhibitory assay)

The anti-diabetic activity was assayed by α-amylase inhibitory assay according to the 3.5-dinitrosalicylic acid (DNSA) method for calculating the percentage of α-amylase inhibition (%) using Acarbose as a standard drug. In brief, 0.5 ml of test solution was mixed with 0.5 ml of α-amylase solution (0.5 mg/ml) with buffer (Na_2HPO_4/NaH_2PO_4 (0.02 M), NaCl (0.006 M) at pH 6.9) to give concentrations ranging from 25 to 800 μg/mL. The mixture was incubated at room temperature for 10 min and 200 μL of the starch solution (1% in water (w/v) buffer (Na_2HPO_4/NaH_2PO_4 (0.02 M), NaCl (0.006 M) at pH 6.9)) was added. The reaction was terminated by adding 200 μL DNSA (coloring) reagent (12 g of sodium potassium tartrate tetrahydrate in 8.0 mL of 2 M NaOH and 20 mL of 96 mM of DNSA solution). At this time, the test tubes were placed in a boiling water bath (100 °C) for 10 min

and the mixture was cooled to ambient temperature and was diluted with 5 mL of distilled water, and the absorbance was measured at 540 nm using a UV-Visible spectrophotometer.

The blank with 100% enzyme activity was prepared by replacing the plant extract with 200 µL of the buffer. A blank reaction was similarly prepared using the plant extract at each concentration in the absence of the enzyme solution. A positive control sample was prepared using acarbose and the reaction was performed similarly to the reaction with plant extract as mentioned above. Absorbance of blank (buffer instead of analyte and amylase solution) and control (buffer instead of extract) samples were also determined. Acarbose was used as standard drug. Percentage of α-amylase inhibition was calculated using the following equation: The α-amylase activity inhibition %:

$$[(Ab_{control} - Abc_{blank}) - (Ab_{sample} - Abs_{blank})/(Ab_{control} - Abc_{blank})] \times 100$$

Where, $Ab_{control}$: absorbance of control; Abc_{blank}: absorbance of control blank; Ab_{sample}: absorbance of sample; and Abs_{blank}: absorbance of sample blank.

Anti-Alzheimer's activity (acetylcholinesterase (AChE) enzyme inhibition)

The anti-Alzheimer's activity was measured by calculating the percentage of acetylcholinesterase (AChE) enzyme inhibition (%) according to Ellman's method. The tested compounds were dissolved in a 0.1 M phosphate buffer, pH 8. To a flat bottom 96-well plate, typical run consisted of, 5 µl of Acetylthiocholine (ATCh) (0.5 mM), 5 µl of 5, 5′-dithiobis-2-nitrobenzoic acid (DTNB) (0.03 mM) and 5 µl of the tested samples solution at the different concentrations evaluated, which were mixed and incubated for 10 min at 30 ^{o}C. Then, 5 µl of AChE (0.3 U/ml) solution was added to the initial mixture to start the reaction and then absorbance was determined at 412 nm. A control run contained all the aforementioned constituents with exception of the test extract. All experiments were performed in triplicates. The concentration of the tested extract that inhibited the hydrolysis of substrate ATCh was determined by linear regression analysis.

Anti-arthritic activity

Protein denaturation

The percentage of protein denaturation inhibition was calculated according to the method suggested by Lavanya *et al* and modified by Soni and Sureshkumar. All results were compared with the standard (diclofenac sodium) that was prepared according to the method described. The control represents 100% protein denaturation. The test control solution (0.5 ml) consists of 0.45 ml of bovine serum albumin (5% w/v aqueous solution) and 0.05 ml of distilled water. The product control (0.5 ml) consists of 0.45 ml of distilled water and 0.05 ml of the test solution. The standard solution (0.5 ml) consists of 0.45ml of bovine serum albumin (5% w/v aqueous solution) and 0.05 ml of diclofenac sodium. Various concentrations (100, 150, 200 $mgml^{-1}$) of test solution and diclofenac sodium (standard) were taken. All the above solutions were adjusted to pH 6.3 using 1 N HCl. The samples were incubated at 37 ^{o}C for 20 min and the temperature was increased to maintain the samples at 57 ^{o}C for 3 min. After being cooled, 2.5 ml of phosphate buffer was added to the above prepared solutions. The absorbance was measured using a UV-Visible spectrophotometer at 416 nm. The control represents 100% protein denaturation. The results were compared with diclofenac sodium. The percentage inhibition of protein denaturation can be calculated.

Proteinase inhibitory activity

This assay was carried out by calculating the percentage of the proteinase inhibitory activity was according to the method suggested. The test was performed according to the modified method of Oyedapo and Famurewa. The reaction mixture (2 ml) contained 0.06 mg trypsin, 1 ml of 20 mM Tris HCl buffer (pH 7.4) and 1 ml of the test sample of different concentrations. The mixture was incubated at 37 ^{o}C for 5 min and then 1 ml of 0.8% (w/v) casein was added. The mixture was incubated for an additional 20 min. 2 ml of 70% perchloric acid was added to terminate the reaction. The cloudy

suspension was centrifuged and the absorbance of the supernatant was measured at 210 nm against buffer as the blank. The experiment was performed in triplicate to confirm the reproducibility of the result. The percentage inhibition of proteinase inhibitory activity was calculated.
